# Quantitative investigation into methods for evaluating neocortical slice viability

**DOI:** 10.1186/1471-2202-14-137

**Published:** 2013-11-06

**Authors:** Logan J Voss, Claudia van Kan, James W Sleigh

**Affiliations:** 1 Anaesthesia Department, Waikato District Health Board, Hamilton 3240, New Zealand; 2 University of Amsterdam, Amsterdam 1012 ZA, The Netherlands; 3 University of Auckland Waikato Clinical School, Hamilton 2340, New Zealand

**Keywords:** Neocortical slice, Seizure-like event, Viability, Electrophysiology

## Abstract

**Background:**

In cortical and hippocampal brain slice experiments, the viability of processed tissue is usually judged by the amplitude of extracellularly-recorded seizure-like event (SLE) activity. Surprisingly, the suitability of this approach for evaluating slice quality has not been objectively studied. Furthermore, a method for gauging the viability of quiescent tissue, in which SLE activity is intentionally suppressed, has not been documented. In this study we undertook to address both of these matters using the zero-magnesium SLE model in neocortical slices.

**Methods:**

Using zero-magnesium SLE activity as the output parameter, we investigated: 1) changes in the pattern (amplitude, frequency and length) of SLE activity as slice health either deteriorated; or was compromised by altering the preparation methodology and; 2) in quiescent tissue, whether the triggering of high frequency field activity following electrode insertion predicted subsequent development of SLE activity — and hence slice viability.

**Results:**

SLE amplitude was the single most important variable correlating with slice viability, with a value less than 50 μV indicative of tissue unlikely to be able to sustain population activity for more than 30–60 minutes. In quiescent slices, an increase in high frequency field activity immediately after electrode insertion predicted the development of SLE activity in 100% of cases. Furthermore, the magnitude of the increase in spectral power correlated with the amplitude of succeeding SLE activity (R^2^ 40.9%, p < 0.0001).

**Conclusion:**

In conclusion, the findings confirm that the amplitude of population activity is a suitable field potential parameter for judging brain slice viability — and can be applied independent of the mechanism of tissue activation.

## Background

The *in vitro* brain slice preparation has become an indispensable tool for neurophysiological research since its introduction nearly 50 years ago by Henry McIlwain [1]. The popularity of this method stems from its unique balance of ease of use, high controllability of experimental conditions and retained tissue function at molecular, cellular and network levels.

An important issue for slice experimentalists is how best to assess tissue viability from one slice to another — and in so-doing make a sound judgment as to whether to exclude a given sample from experimental testing. This should be of more than just passing interest, because variation in slice condition from trial to trial has potential to greatly confound results. In slice studies in which seizure-like event (SLE) field potentials are the main output, event amplitude is the go-to criterion for inclusion or exclusion [2]. Surprisingly, this practice has been adhered to despite a lack of firm scientific evidence for its justification [3,4].

A separate (but related) issue is how best to judge slice viability in protocols in which SLE activity is intentionally suppressed. There are occasions when experimental protocols require comparison groups in which SLE activity is inhibited [5] ― and there are currently no recognized means of classifying slice viability under these conditions.

In this study we sought to address both of these matters using the zero-magnesium SLE model. Accordingly, the study was divided broadly into two parts. In the first, we characterized the SLE parameter(s) that correlated with deteriorating or compromised slice health. The aim was to determine unequivocally whether SLE amplitude (or an alternative measure) is a reliable means of judging slice condition. In the second, we explored the anecdotal finding that insertion of extracellular electrodes into quiescent tissue sometimes induces a rapidly waning burst of high frequency activity in the field potential recording. We hypothesized that appearance of this high frequency activity reflects the response of healthy tissue to the trauma of electrode insertion ― and could be used to evaluate slice viability when SLE activity has been suppressed.

## Results

**Figure 1:**
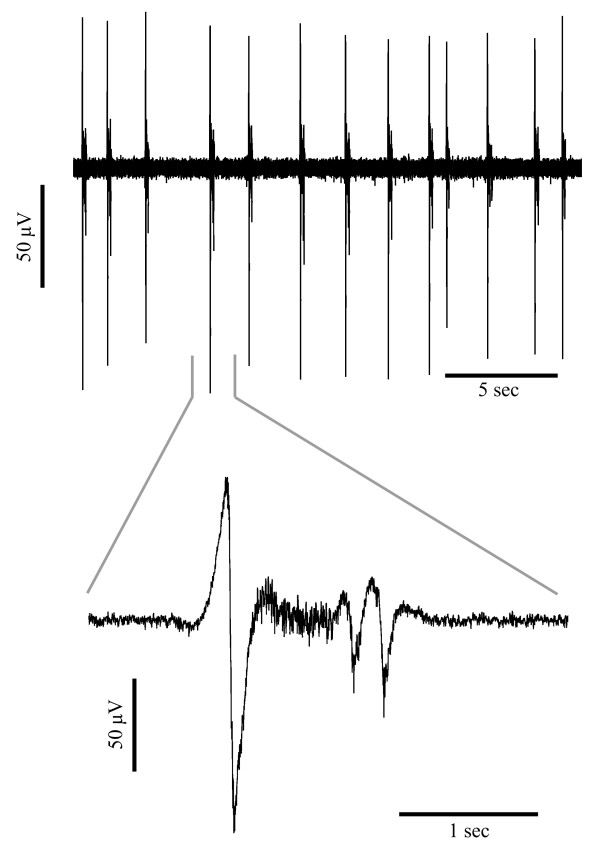
Example of the pattern of zero-magnesium SLE activity recorded from one slice showing a compressed time view (top) encompassing multiple SLEs and an expanded view (bottom) of a single SLE.

An example of the pattern of seizure-like event extracellular field potential activity induced by removal of magnesium from the artificial cerebrospinal fluid (aCSF) is shown in Figure 1. The seizure-like bursts are typical of those recorded under zero-magnesium conditions.

### Pattern of SLE activity in deteriorating/compromised slices

SLE activity changed variably over time as the tissue deteriorated (Table 1). While event amplitude, frequency and length all trended downwards over time, the only significant change was a reduction in event amplitude. The data indicate that SLE amplitude of less than 50 μV is indicative of tissue that is poorly viable and may not be able to sustain population activity for more than 30–60 minutes. However, it is important to note that this is a generalization based on variable data. For example, in two cases, SLE amplitude was greater than 200 μV within 30 minutes of all activity ceasing. Furthermore, in one slice SLE amplitude at the beginning of the recording was 10 μV but continued to deliver stable activity for upwards of 7 hours.

**Table 1:** SLE characteristics as neocortical slice tissue deteriorated from 1 hour after starting recording (Seg 1) to 1–2 hours before all activity stopped (Seg 2) to 30 minutes before all activity stopped (Seg 3)

	**Frequency (/min)**	**Amplitude (μV)**	**Length (s)**
**Seg 1**	6.4(2.5-25.8)	126.4(9.2-1174.6)*	1.0(0.4-1.4)
**Seg 2**	4.5(1.3-13.2)	55.0(15.9-1466.6)	1.1(0.3-1.3)
**Seg 3**	3.7(1.8-18.0)	25.6(7.5-252.1)	0.7(0.3-1.2)

*p < 0.01, compared to Seg2 and Seg3 (Friedman Test, Dunn’s Multiple comparisons).

Data are median(range).

The alterations made to the slice preparation methods were designed to deliberately undermine tissue condition ― with the aim of establishing unequivocally whether low SLE amplitude correlated with poor slice viability. The results were clear in showing that all three interventions resulted in significantly lower SLE amplitude compared to our “standard” protocol (Table 2). The data confirm that a peak-to-peak amplitude value of approximately 50 μV or less is a good indication that tissue condition has been compromised. With the exception of a lower SLE frequency in the tap water group, event amplitude was the only variable affected. Under the conditions tested in this study and for the analysis methods employed, we can conclude that event amplitude is the single most useful variable for assessing slice viability.

**Table 2:** Median (range) SLE characteristics using preparation methods designed to impair tissue viability

	**Frequency (/min)**	**Amplitude (μV)**	**Length (s)**	**% Active locations**
**“Standard” conditions**	**3.2(0.6-11.0)**	**173.0(32.2-1247.1)**	**1.7(0.6-8.7)**	**57.5**
Long CO_2_	2.5(0.5-11.1)	52.8(9.9-840.1)**	2.0(1.1-5.1)	60.3
Slicing delay 30 mins	2.5(0.6-9.7)	63.1(13.2-236.5)**	2.0(1.4-3.7)	40.8
Unfiltered tap water	2.0(0.6-31.8)*	36.0(10.3-424.7)**	1.8(0.7-3.3)	58.9

“Standard” conditions are defined in the methods section.

*p = 0.0039, compared to “Standard” conditions (Mann–Whitney).

**p = 0.0001, compared to “Standard” conditions (Mann–Whitney).

### Quiescent tissue viability correlates

**Figure 2:**
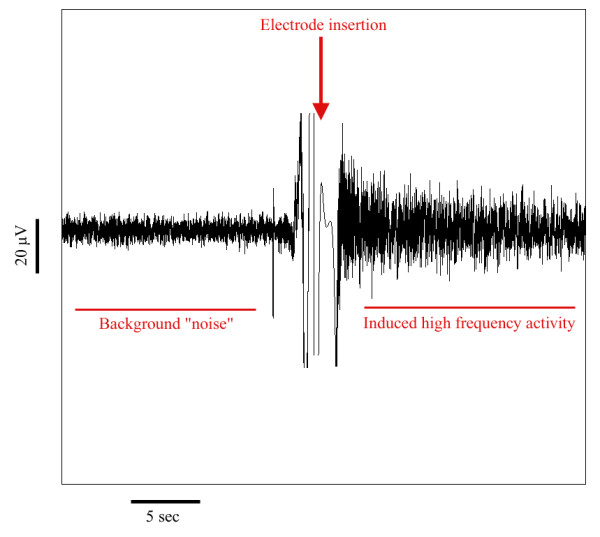
Example showing the increase in high frequency field potential activity triggered by electrode insertion into the tissue.

**Figure 3:**
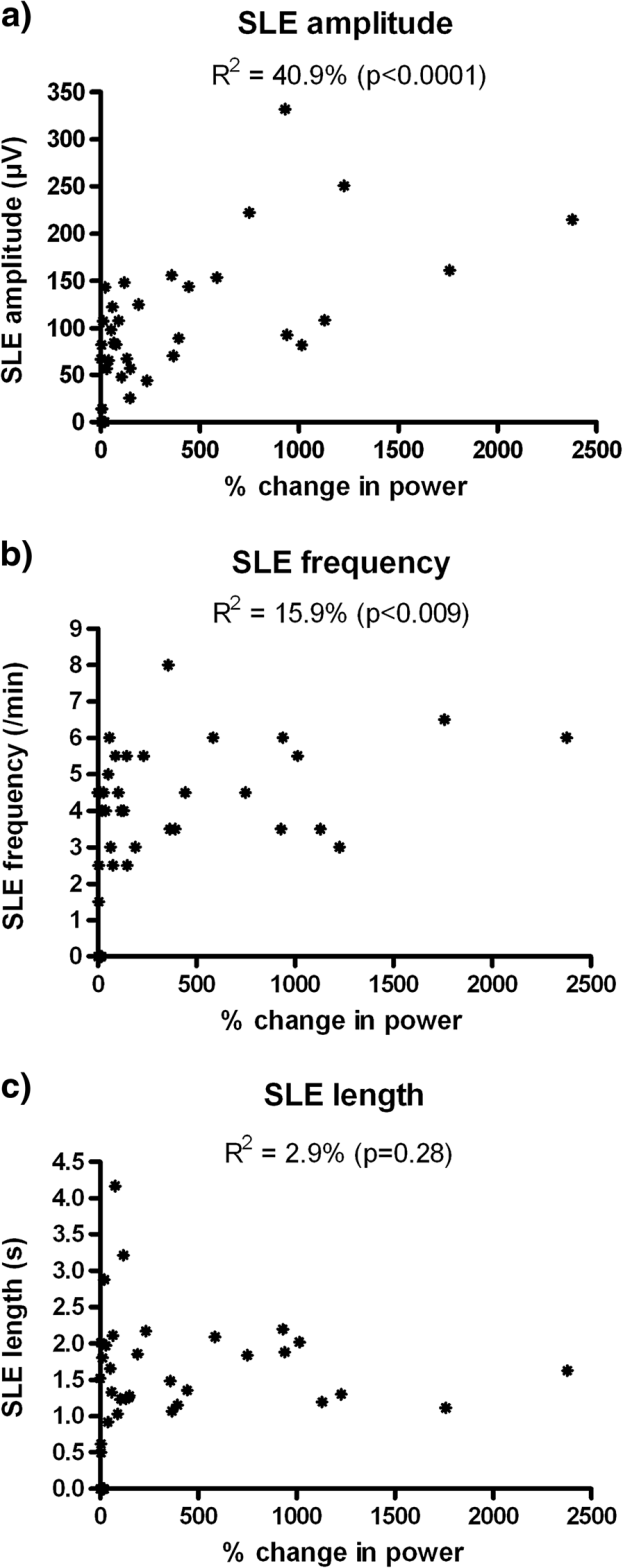
Correlations of SLE amplitude (a), frequency (b) and length (c) with the% change in high frequency spectral power triggered by electrode insertion into the tissue.

An example of the pattern of activity induced by electrode placement into quiescent tissue is shown in Figure 2. Of the 42 recordings, SLEs developed in 36 cases. In these, the mean(SD) change in spectral power following electrode insertion was 382.7(554.5)%; compared to 5.7(12.4)% in the 6 “non-responders”. The difference is statistically significant (p = 0.004, Mann–Whitney test). SLEs occasionally (8/42) developed even though no noticeable change (judged by eye to be a change of <20%) in high frequency power was observed, but the opposite was never true. That is, SLEs developed in 100% of instances where the increase in power was >20% (28/42). Furthermore, the greater the spectral power increase, the larger the amplitude of the SLEs that subsequently developed (R^2^ 40.9%, p < 0.0001); and (to a lesser extent), the higher the SLE frequency (R^2^ 15.9%, p < 0.009) (Figure 3). SLE frequency was also positively correlated with the length of time the slices were held in normal aCSF prior to recording, which may account for some of the variability in SLE frequency outcome. There was no correlation between normal aCSF hold time and either SLE amplitude (R^2^ 0.2%, p = 0.78) or SLE length (R^2^ 0.005%, p = 0.97).

When slice viability was intentionally compromised (n = 6, from one animal), the change in spectral power following electrode insertion was 5.1(-7.7-126.5)%, compared to 82.9(-19.5-2378.6)% under standard conditions (p = 0.036, Mann–Whitney test). In the compromised group, SLEs developed in only 50% of cases, compared to 86% under standard slicing conditions — with a median(range) amplitude of 13.7(0–54.9) μV and 82.1(0–332.2) μV, respectively (p = 0.02, Mann–Whitney test) ― confirming the relationship between slice viability, the tissue response to electrode insertion and SLE development.

## Discussion

In this study we addressed two questions relating to the evaluation of cortical brain slice viability. Firstly, when recording SLE activity under zero-magnesium conditions, is event amplitude a valid method for judging the health of the slice? Event amplitude is widely used to evaluate slice condition at the beginning of an experiment, with many researchers applying amplitude-based exclusion thresholds [2,4]. The logic is sound, because a greater number of viable cells will be potentially recruitable for each event in a healthier slice. However, the amplitude of population activity is not governed solely by the number of active units, but also the synchrony of those units [6]. Thus, lower amplitude activity does not by a priori mean fewer viable cells. It’s surprising then that examination of the literature uncovers scant objective investigation of this topic. In this study, we approached the subject by quantifying SLE characteristics following interventions designed to be detrimental to tissue health. The group comparisons confirmed that compromised tissue tended to produce SLEs of lower amplitude. Furthermore, the other measures of SLE activity did not associate with slice condition, validating event amplitude as the most suitable parameter. As a caveat to this, it should be noted that the downward trend in event amplitude may be more informative than the absolute value, making the choice of threshold for slice exclusion/inclusion more difficult.

Secondly, we were interested in whether the tissue response to electrode penetration could predict subsequent SLE development; and hence identify healthy slices in which SLE activity had been intentionally suppressed. The results paralleled those of the first part of the study, showing that the amplitude (power) of the high frequency response to electrode-induced tissue trauma correlated with the amplitude of SLE activity recorded from the same slice. In other words, the amplitude of field potential population activity *per se* reflects the underlying health status of the tissue, independent of the method used to generate the activity.

In this study we used the zero-magnesium model of epileptogenesis. This method has been widely used for investigating mechanisms of epilepsy [7,8] and has also been applied to the study of anaesthetic drug effects [9,10]. The model therefore has wide applicability in the field of neurophysiology. Moreover, the two complementary facets of this study suggest that the findings can be extrapolated to other slice models involving different methods of tissue activation.

## Conclusions

In conclusion, the amplitude of population activity ― independent of its mechanism of production ― is a robust electrophysiological measure of neocortical slice viability.

## Methods

### Ethics statement

The Waikato Ethics Committee at Waikato University, Hamilton, New Zealand approved all experimental procedures (approval #859).

### Artificial cerebrospinal fluid solutions

Unless otherwise stated, the solutions were made with double distilled water and stored at 1–4°C for no longer than 7 days. All solutions were bubbled with carbogen (95% O_2_; 5% CO_2_) for at least 15 minutes before use. Three solutions were used as follows (amounts in mM):

1) *“Protective”* aCSF for brain extraction and tissue slicing: NaCl 92.7, KCl 3, MgCl_2_ 19, NaH_2_PO_4_ 1.2, NaHCO_3_ 24 and D-glucose 25 [11].

2) *“Normal”* aCSF for brain extraction and tissue slicing: NaCl 125, KCl 2.5, MgCl_2_ 1, CaCl_2_ 2, NaH_2_PO_4_ 1.25, NaHCO_3_ 26 and D-glucose 10.

3) *“Zero-magnesium”* aCSF lacking magnesium ions: NaCl 124, KCl 5, CaCl_2_ 2, NaH_2_PO_4_ 1.25, NaHCO_3_ 26 and D-glucose 10.

The composition of solutions 2) and 3) were designed to ensure equal osmolarity, which was confirmed using an RE388TX conductivity meter (EDT Instruments Ltd, UK), at a frequency selected by the meter such that polarization effects were minimized. The conductivities of the two solutions were 1.578 S m - 1 and 1.558 S m - 1, respectively.

### “Standard” procedures for tissue preparation

Adult male and female wild-type (129SV) mice were used to obtain neocortical brain slices. Prior to decapitation and brain dissection, the mice were anaesthetized with carbon dioxide (approximately 30 s exposure) until unresponsive to paw pinch. The brain was freed from adjacent tissue and placed into ice-cold *“Protective”* aCSF. The delay between brain removal and slicing was not more than 10 minutes. Immediately before slicing, the anterior and posterior 1–2 mm of the cerebral cortex was removed with a razor blade. The tissue block (between Bregma -2 to -5) was glued to a stainless steel stage for slicing into 7–9 400 μm thick coronal sections using a vibratome (Campden Instruments, UK). Slices were held in zero-magnesium aCSF solution to recover for at least an hour and a half prior to recording at room temperature (22–27°C). For experimental recording, slices were individually transferred to a submersion-style recording bath (Tissue Recording System, Kerr Scientific Instruments, New Zealand) perfused with zero-magnesium aCSF solution at a gravity-fed flow rate of 5.0 ml/min.

### Extracellular field potential recording

SLE activity (see Figure 1) was recorded using a 50 μm Teflon-coated tungsten electrode. The electrode was referenced to a silver/silver-chloride disc electrode positioned in the recording bath, which also served as the bath ground. The data was recorded with a 1000× gain, low- and high pass filtered at 3000 Hz and 1.0 Hz respectively (Model 1800 AC amplifier, A-M Systems, USA). The data was collected using LabChart6 and Spike2 and saved for later analysis using Matlab (version 7.13.0.564, R2011b, The MathWorks Inc., Natick Massachusetts USA).

### Experimental procedures 1: pattern of SLE activity in compromised slices

This section was divided into two parts. In the first, slices were established in the recording bath as above and stable SLE activity identified. No minimum amplitude threshold was applied because we made no assumptions about the correlation between SLE amplitude and slice viability. The aCSF solution flow was then stopped and the recording continued until all SLE activity had stopped. On occasions this required recordings to be continued overnight to be sure all activity had ceased. Each recording was subsequently analysed at 3 time-points for SLE frequency, length and amplitude: 1) within 1 hour of establishing the recording (seg1); 2) 1–2 hours before all activity stopped (seg2) and; 3) within 30 minutes of all activity stopping (seg3). The aim was to identify changes in SLE activity as the slice deteriorated, thereby providing an objective measure of slice health. Data was collected from 11 slices (from 4 animals).

In the second, we changed aspects of the slice preparation methodology so as to compromise tissue outcome. The aim was to validate, under varying conditions, the outcomes from the first part of the study. The following changes were made to the standard methodology as described above and SLE outcome comparisons made.

1) Longer (2 minutes, complete asphyxiation) CO_2_ anaesthesia for mouse decapitation (n = 38, 6 animals)

2) Whole brain held in “brain extract” solution prior to slicing for a longer period (30 minutes) (n = 23, 4 animals, respectively)

3) Unfiltered tap water used for aCSF solutions (n = 33, 5 animals)

Once established in the recording bath, four locations of equidistant separation (two from each hemisphere) were sequentially recorded from each slice. The amplitude, frequency and length of SLE activity were averaged over 5 consecutive events from each location. If no SLE activity was evident at any one location, this was noted as a non-active site. Non-activity was defined as: 1) no SLEs observed within 3 minutes of establishing the recording or; 2) less than 5 SLEs recorded within 10 minutes of establishing the recording. The total proportion of active locations was calculated for each condition.

### Experimental procedures 2: viability testing in SLE-quiescent tissue

Mouse cortical slices were prepared as per the standard procedures outlined in section 2.3, with the exception that the slices were maintained for the 1.5 hour post-slicing period in *normal aCSF* to prevent SLE development. In total, 16 slices were prepared from 2 animals, giving 42 recordings. For comparison, tissue preparation in a separate group (6 slices from 1 animal) was intentionally compromised by delaying slicing of the extracted brain by 60 minutes.

Thereafter, each slice was transferred to the recording bath perfused with zero-magnesium aCSF as described. 1–3 tungsten electrodes (50 μm) were positioned at arbitrary locations within the cerebral cortex. At least 10 s of noise-free baseline recording was made prior to electrode insertion for comparison with the 10 s immediately after electrode insertion. Thereafter, zero-magnesium aCSF perfusion was continued for 40 minutes at a constant flow rate and no other interventions made. Each recording was saved for later off-line analysis of the correlation between electrode-induced activity and subsequent SLE development (if any).

### Statistical analysis

Since not all data was normally distributed (Kolmogorov- Smirnov test), non-parametric tests were used for all comparisons and data presented as median (range). The Mann–Whitney test was used for 2-sample analyses. For multiple comparisons a p-value <0.008 was considered significant, adjusted according to Bonferroni’s correction. Otherwise, a p-value <0.05 was considered statistically significant. Electrode-induced activity was calculated as the change in summed power in the 10–50 Hz frequency band over the 5 s immediately before and after electrode insertion. This was then correlated with the pattern of SLE activity that subsequently developed; namely the peak in SLE amplitude, frequency and length. Because quantification of SLE length is difficult to automate, this parameter was derived from the manual selection of 5 sequential SLEs at the end of the recording. If no SLEs developed, all parameters were given values of zero.

## Abbreviations

SLE: Seizure-like event; aCSF: Artificial cerebrospinal fluid.

## Competing interests

The authors declare that they have no competing interests.

## Authors’ contributions

CVK carried out the experiments and data analysis. LJV helped carry out the experiments and data analysis; and drafted the paper. JWS helped with editing the manuscript. All authors read and approved the final manuscript.
